# The Effect of Top-Level Domains and Advertisements on Health Web Site Credibility

**DOI:** 10.2196/jmir.6.3.e24

**Published:** 2004-09-03

**Authors:** Joseph B Walther, Zuoming Wang, Tracy Loh

**Affiliations:** ^1^Department of CommunicationCornell UniversityIthaca NYUSA

**Keywords:** Internet, credibility, Web sites, domains, advertising

## Abstract

**Background:**

Concerns over health information on the Internet have generated efforts to enhance credibility markers; yet how users actually assess the credibility of online health information is largely unknown.

**Objective:**

This study set out to (1) establish a parsimonious and valid questionnaire instrument to measure credibility of Internet health information by drawing on various previous measures of source, news, and other credibility scales; and (2) to identify the effects of Web-site domains and advertising on credibility perceptions.

**Methods:**

Respondents (*N* = 156) examined one of 12 Web-site mock-ups and completed credibility scales in a 3 x 2 x 2 between-subjects experimental design. Factor analysis and validity checks were used for item reduction, and analysis of variance was employed for hypothesis testing of Web-site features' effects.

**Results:**

In an attempt to construct a credibility instrument, three dimensions of credibility (safety, trustworthiness, and dynamism) were retained, reflecting traditional credibility sub-themes, but composed of items from disparate sources. When testing the effect of the presence or absence of advertising on a Web site on credibility, we found that this depends on the site's domain, with a trend for advertisements having deleterious effects on the credibility of sites with .org domain, but positive effects on sites with .com or .edu domains.

**Conclusions:**

Health-information Web-site providers should select domains purposefully when they can, especially if they must accept on-site advertising. Credibility perceptions may not be invariant or stable, but rather are sensitive to topic and context. Future research may employ these findings in order to compare other forms of health-information delivery to optimal Web-site features.

## Introduction

One of the most interesting aspects of the diffusion of the Internet is its use by millions to access and to discuss information related to health and medicine. Surveys estimate that 55% of Americans with Internet access seek health information online [1]. This phenomenon prompts both praise and concern. Praise, because individuals can access information to help them address physical and psychological maladies at any time for no cost, once they have computer access. Concern, especially from health professionals, that information freely distributed online is subject to no professional authorization and there is no way of verifying the credentials of those who post the information; thepossibility exists for amateurs to misinform one another, with harmful consequences. The credibility of health information on the Internet is also a concern to users themselves. Eighty-six percent of health-information seekers are concerned that online sources are unreliable. Fifty-two percent of users who have visited health sites think that "almost all" or "most" health information that they see on the Internet is credible, and 44% think that they can believe only "some" online health information. Fifty-eight percent of health**-**informationseekers checked to see who was providing the information on the Web sites they visited [1].

Some steps have been taken in order to address this concern. Bona fide medical organizations such as the American Medical Association have formulated Web-site design recommendations intended to facilitate understanding and certify authenticity [2]. Pharmacies hand out lists of questions (oddly, without answers) that Web surfers should ask when perusing medical Web sites to help them decide about the utility and authenticity of information online. In some cases, medical experts rated the content of health-related Internet sources; in one trial they rated information as poor to potentially dangerous, although Craigie et al [3] found that the experts "showed a low agreement when rating the postings." A meta-analysis by Turow and his colleagues [4] revealed, among medical and academic researchers alike, "a startling lack of consensus among (medical and academic) researchers regarding the meaning of basic terms as quality, accuracy, and depth of detail when it comes to a website."

In contexts other than health and medicine, the credibility of Web sites has been explored, but whether the principles uncovered in e-commerce or other Web transactions apply as well to medical information seeking is an issue that is just beginning to come into focus. Little is actually known about how end-users of online health and medical information evaluate the credibility and utility of such information, and it appears that users themselves have limited awareness of how they find and evaluate Internet-based information on health and medicine [5].

In this study, we examine historical approaches to the study of credibility, the challenges facing the application of these approaches to Internet information, and new aspects related to credibility that Internet channels introduce. We then describe an empirical research project in which we developed a parsimonious instrument to measure how users assess health-information credibility online, and how they evaluate it with respect to different features of health-related Web sites-domain and advertising-to assess whether these attributes affect the credibility users ascribe.

### Credibility

In the last fifty years, credibility has been conceptualized and studied in a variety of ways. Much research has been directed towards studying credibility, mostly in terms of its various sub-dimensions of source, message, and medium credibility. There tends to be considerable overlap between the various dimensions of credibility on which research has focused [6,7,8,9].

#### Source Credibility

Traditionally, credibility research focuses on the question of what makes a communicator believable and persuasive. While analysis of this kind dates back to Aristotle, one of the most important theoretical formulations divided source credibility into the two dimensions of *expertise* and *trustworthiness* [10]. Expertise is defined as a communicator's qualifications and/or ability to know the truth about a topic. Trustworthiness relates to a judgment about the communicator's motivation either to tell the truth about a topic, or to bias information for self-serving motives (such as commercial gain). This dynamic emerges in recent focus group research assessing how consumers search and appraise Internet information about medicines: While some respondents regard pharmaceutical companies as the "official" information source, others prefer government agencies, organizations, and educational institutions as information sources, considering them to be impartial [5].

The history of credibility research suggests that a variety of theoretical dimensions and empirical measures may be relevant to assessing credibility. These dimensions include safety, qualification, and dynamism [11], authoritativeness, sociability, character, competence, composure, and extroversion [12,13], and other similar dimensions [14,15].

In addition, the relationship between the receiver and the source has been identified as an important factor in determining the degree of credibility accorded to the source [16,17]. How Internet users relate to experts may affect credibility assessments. Perception and measurement of source credibility may differ depending upon the type of source being evaluated as well as the context in which the evaluation occurs [14,18,19,20,21].

The factor-analytic approach to defining dimensions of credibility has been criticized on a number of grounds. For instance, characterizations of the research as atheoretical and data-driven have been made. Cronkhite and Liska [18] argued that factor structures depend on a number of aspects, including the rating scale used, the speakers chosen, the raters chosen, and the method of factor analysis. Delia et al [22] found that attitudes toward a source were based more on context-relevant beliefs than on generalized evaluations. In different contexts, different dimensions of evaluation became more relevant.

These concerns both reject the adoption of one set of existing factors over another, and highlight the need to identify the relevant factors based on the content and audience for each research setting, an approach that has also been adopted in the present study.

#### Media Characteristics

The concept of credibility has been widely explored in the domain of traditional media, with research investigating the relative believability of particular forms of communication (eg, newspapers vs television), where cross-medium comparisons of credibility dimensions have been regularly examined. To make such comparisons, "the credibility of various media has been measured by comparing perceptions of the believability, accuracy, fairness, bias, trustworthiness, ease of use, completeness, reliability, or attractiveness, for example, of the media themselves…" [23]. For instance, some studies on the credibility of print and television reveal that television is more believable than print media; others demonstrate that only newspapers are especially credible when compared to magazines and other print media (for review, see [24]).

Gaziano and McGrath [25] developed a 12-item media credibility scale, comprising the following items: is fair, is unbiased, tells the whole story, is accurate, respects people's privacy, watches out for the public's interest, is concerned about the community's well-being, separates facts from opinions, can be trusted, is concerned about the public's interest, is factual, and has well-trained reporters. Meyer [26] reduced the scale to five items**:** fairness, bias, completeness, accuracy, and trust.

#### Internet Credibility Issues

The Internet's ability to combine aspects of and collapse barriers between traditional source, message, and media studies has opened up new vistas in credibility research. The Internet is a uniquely versatile medium of numerous communication and information functions and ought to be treated as such [27]. As traditional forms converge, new measures of credibility arise in addition to the numerous measures already established.

Recently, Sundar and Nass [28] experimentally examined how people identified and evaluated sources of news, all of which came through Internet channels. Subjects were exposed to news stories presented by computers and appearing to be transmitted via the Internet. Subjects were led to believe that the stories were chosen by a corporate news organization, by the computer, by a peer discussion group, or by the subject himself or herself. While there were no differences on a measure of credibility, significant differences on other measures suggest that computer users make distinctions about information quality, and prefer different information sources, based on the institutional nature of the source.

#### Web Credibility

The convergence of genres of information via the Web makes it problematic to assess online credibility. As Metzger et al [23] pointed out, Web-site expertise can be reflected in the site's informativeness, the display of the appropriate credentials, the site sponsor's reputation, or the type of site sponsor (ie, institutional vs individual). *Trustworthiness* may be communicated through explicit policy statements or a lack of advertising and commercial content; and attractiveness or dynamism can be presented through dimensions of the Web site's appearance (eg, layout, graphics, font, color, etc). According to Eastin [29], *dynamism* also plays a key role in perception of online content, which can be affected when a message or a Web site's presentational features are highly dynamic. Fogg et al [30] found that commercial associations (eg, more advertisements) and a feeling of amateurism (eg, broken links) decreased credibility, while a real-world feel (eg, a physical address listed in site), perceived integrity (eg, explicit policy statement), and tailoring (eg, site sends emails confirming transactions) can increase credibility. Novel credibility concerns may also arise when evaluating Web sites. For instance, issues of security, consumerism, and usability, which usually are not the concerns of the traditional media credibility, arise in various Web contexts.

#### Medical Information

In the specific domain of health information on the Web, a few studies are notable. Following arguments about variable attention to content cues versus heuristic cues as a function of topical knowledge, Eastin [29] explored the same dynamics in users' evaluation of online health information. Eastin's study experimentally varied source expertise (high, medium, low) and subjects' knowledge about the topic (HIV vs syphilis) among a sample of college students. Participants tended to rate all information as relatively credible, with effects obtaining for content knowledge and source expertise. There was no interaction effect among these variables.

Eysenbach and Köhler [31] examined what characteristics of health Web sites users purported to use in evaluating credibility, and also observed the discrepancy in subjects' actual search and evaluation behaviors. Users indicated a variety of symbols that would enhance believability in online health information, including the scientific or institutional source of the information, site owner credentialing, and content updating. In their actual search behavior, however, users almost entirely neglected such resources, relying on search engines (and top-to-bottom ordering of search results) to select sites to browse, spent a median of 37 seconds on a site, and remembered the domain of the sites from which they gleaned information only 23% of the time. These results from the health domain mirror more general tendencies for users not to check or verify the veracity of Web information in other kinds of research [32].

Dutta-Bergman [33] recently found that the completeness of information affected attitudes of health-information users. While credibility was not a specific construct of concern in this study, the outcome of credibility-persuasion-was. Dutta-Bergman offered two levels of information completeness and argument quality on experimental sites offering heart-and-diet information. The completeness variable affected the attitudes of both casual readers and readers prompted to imagine they had heart disease.

### Health Care and the Internet

According to Pew Research, the Internet is being used by many Americans (55% of those with Internet access) to gain health or medical information. Seventy percent of those who said they have been swayed by what they read online the last time they sought health information said that the information they obtained online influenced their decision about how to treat an illness or condition, 50% said that the information led them to ask a doctor new questions or get a second opinion from another doctor, and 28% said that the information they found online affected their decision about whether or not to visit a doctor.

#### Hypotheses

Given the lessons of the factor analytic approaches to credibility, we needed to develop a parsimonious and appropriate scale with which to measure credibility in the context of online health information, and to assess it using adults who were actual or prospective health-information users. Second, we sought to ascertain what characteristics enhanced or detracted from health-information Web-site credibility. In order to develop an instrument, we collected those measures used in previous research and subjected them to the treatments described below. In order to identify Web-page characteristics affecting credibility, we developed the following hypothesis based on the literature reviewed above:

H1: Different top-level domains (*.org*, *.com*, *.edu*, *.gov*) influence the credibility of the Web site.

Because Web sites with explicit commercial natures are more likely to be associated with greater self-interest, we also posited the following:

H2: The *.com* domain reduces the credibility of a Web site.

H3: The presence of advertisements reduces the credibility of a Web site.

## Methods

Data were collected in two phases using two samples and two parallel sets of stimulus materials.

### Stimulus Materials

A number of health-related Web sites were reviewed for typical features in order to generate plausible-looking Web pages containing the manipulations of interest to this study. Commonalities were noted with respect to the size of the headers and text, and the presence of graphics depicting couples or individuals who appeared to be doctors. Typical-looking Web-page mock-ups were created that resembled many of these sites' home pages.

**Figure 1:**
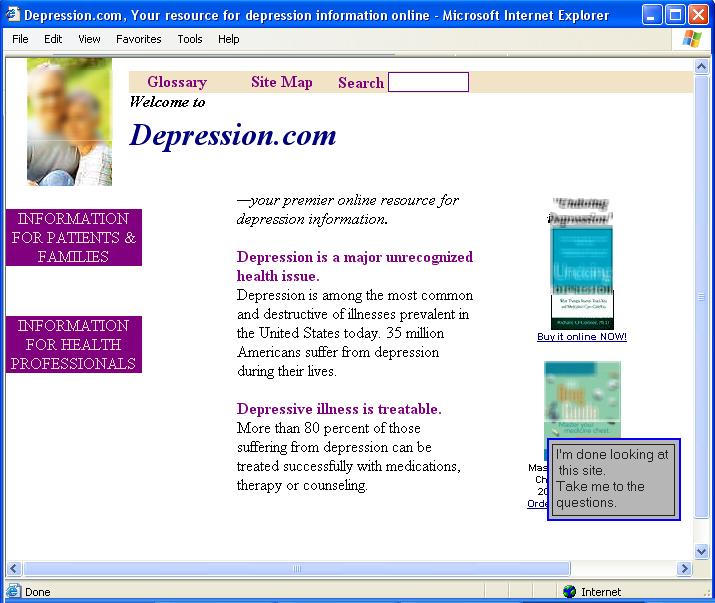
Web site mock-up

The mock-ups varied with respect to operationalizations of several variables. First, two topical health issues were identified for use as examples in this research on the basis of their popularity as online health topics, both as Web-related information sources and as subjects of peer (Usenet) discussion topics: arthritis and depression [34]. Second, the headers were varied to reflect differences in the following domain types: arthritis.com, arthritis.edu, arthritis.gov, and arthritis.org (with parallel differences for depression). Third, within each of these conditions, half the mock-ups featured advertisements, in this case, for consumer-level pharmacological books, while the other half did not. All of the mock-ups featured a photo copied from an actual health-information Web site depicting a smiling middle-aged couple, which was common on such sites, in order to make the mock-ups look like typical Web pages of this nature. All mock-ups contained the same text. Thus the stimulus conditions comprised variations according to a 2 (topic) x 4 (domain) x 2 (advertising/no advertising) design, resulting in 16 different versions. A sample is featured in Figure 1, but for purposes of publication, the photo and advertisment titles have been blurred.

### Measures

Although credibility has been conceptualized and measured in many ways in various contexts, it is not clear whether the dimensions and operationalizations of credibility in previous research provide the most parsimonious and applicable dimensions of this construct for the specific domains of health communication via the Internet [23]. In order to discern the most applicable measures, which was a major focus of this study, a number of existing measures were collected that bore some conceptual connection to one aspect or another of online health-information credibility. These existing measures included source credibility [11,12], and news credibility (excluding items specifically referencing reporters [25]). All of these measures exist as semantic differential items.

Items from all of these measures were gathered on one questionnaire and arrayed on a common 7-interval scaling. Items that were duplicated from different sources were used only once.

### Participants and Procedures

For research related to health issues it is often important to obtain a sample that has a wider age range than typically is found among college students. Extending from principles of elaboration likelihood theory [35], adults who are more likely to be concerned about health issues on a first- or second-hand basis will attend to features of a presentation differently than will younger and presumably healthy individuals whose experience with health topics is less personal. In order to attract a sample of adult respondents, researchers employed an intercept survey technique at a local shopping mall in a northeastern US suburban city for several weekends in November, a busy holiday shopping season. Researchers were asked to limit their activity to a relatively constrained location in the mall, from which they approached passersby who appeared to be greater than college aged.

Stimulus materials were printed as color copies on paper. When a researcher approached a passerby and the prospective respondent indicated willingness to be questioned, researchers screened participants using several questions about whether they had ever used the Internet, had an email address, or used online discussion systems. Negative responses to these qualifying questions terminated the intercept, and the prospective respondent was thanked and dismissed. Upon qualification, participants were asked to examine one of the 16 versions of the mock-up Web page, which were randomly distributed. Each participant was asked to examine the top page-the mock-up-for as long or as short a time as he or she wished, and then to turn to the subsequent pages to complete a self-administered questionnaire. Participants were asked not to turn back to the first page after moving on. Participants were offered a place to sit at a table and a confection if they wished.

The first item on the questionnaire asked respondents to write down the name of the Web site the home page of which they had just seen. This question was used in order to track participants' awareness of the domain name, although it was unclear at the outset how much difference there might be in their overall responses due to their cognizance of domain type. Following this item, the credibility items were presented for self-administered completion, with demographic items at the end of the questionnaire.

The first phase of data collection yielded 111 participants, with a median age of 32, 46% of whom indicated they were male, and 53% female.

In order to increase the sample size for more robust analysis, a second phase of data collection was undertaken. A different strategy, more efficient than the field intercept method, was used in this phase to attract an adult sample. In this phase, a snowball sampling strategy was employed: Students taking an introductory communication course were addressed in class and sent an email message that they were asked for forward to their parents, who in turn were asked to participate in the research. The email message contained a URL for a Web page, which introduced the purpose of the study, instructed participants that they would soon see a home page for a Web site that they might examine for as long or as short a time as they wished, and told them they would be asked to answer a series of questions if they clicked a button to continue. When they clicked this button, a JavaScript routine randomly redirected the participant to one of 16 versions of a Web-site mock-up. These mock-ups were identical to those used in the paper version of the study in phase 1. However, another button was made to float over the site content so that, no matter where on the page the participant might scroll, the prompt to click to move to the questionnaire was always present. When participants clicked this button, the same semantic differential items were presented using a Web form, with radio buttons for each scale on which to record responses. Participation was anonymous in every way. This sample yielded 45 individuals, with a median age of 50 and the gender composition of 68% female. Concerns over the possible differences in participant responses due to the two data collection methods are addressed in the hypothesis test results, below.

## Results

### Scaling

The first objective of this study was to create a reliable and parsimonious measure for Web credibility related to online health information. Data from the questionnaire were subjected to a principal component factor analysis with Varimax rotation to identify the items and dimensions of online health-information credibility from otherwise disparate but potentially overlapping measures. In order to identify the most parsimonious measure, we employed very conservative criteria for selecting a factor solution: (1) All factors had to have eigenvalues of 1.5 or better; (2) the Scree test indicated reasonable incremental improvement in variance accounted for by the addition of a given factor; (3) all retained factors had to contain at least three items with primary loading of 0.60 or better and secondary loadings below 0.40; (4) among solutions meeting the first three criteria, the one accounting for the most variance was to be selected. Initial results show up to six factors with eigenvalues greater than 1.5, accounting for 60% of the variance. However, after the application of the above criteria, the results indicated an optimal three-factor solution explaining 48.6% of the variance.

After applying these criteria, we conducted cross-tab analysis to examine the discriminating validity of items within each dimension. It is often the case that factor analysis clusters items together that show little variance (ie, scores are not high or low, but huddled around the mid-point of the scales [36]), and such items reduce the overall utility of the measure. Following procedures articulated in Klingle et al (1995), we identified the cut-off scores associated with the bottom quartile (23%) and the top quartile (78%) of the dimensional totals by summing up the values of all items within a dimension. Values in the scale below 23% were coded as 1, and values above 78% were coded as 2. Then for every item, we calculated the item-specific cut-offs by treating 5-7 scores as high and 1-3 as low. If the score on the original item was 3 or below, it was coded as 1, and if its score was 5 or above on the original item, it was coded as 2. Cross tabs were used to test the item-to-scale correspondence, and the phi coefficient was used to see whether a high score on an item also showed a high score on the total. Any item that failed in this aspect was dropped from the scale. Only one item, "friendly," did so, and it was subsequently removed from further analysis.

The first factor appeared to represent *safety* (Cronbach α= .91). *Trustworthiness* is the second factor (α= .82). The last factor is *dynamism* (α = .77). The factor structure and item means and standard deviations appear in Table 1. These dimensions have conceptual overlap with previously articulated credibility dimensions, although the combination of items comprising the factors is unique.

**Table 1:** Factor structure, means, and standard deviations: 16-item measure for Internet health credibility *

Factors and Items	*M*	*SD*	Factor Loadings		
1. Safety
Just/Unjust	4.50	1.415	.787	.302	.097
Friendly/ Unfriendly	5.29	1.332	.771	.128	.149
Safe/Dangerous	4.98	1.421	.762	.309	.114
Kind/Cruel	5.15	1.229	.761	.156	.075
Nice/Awful	4.79	1.259	.737	.289	.001
Good-natured/Irritable	4.80	1.078	.670	.174	.128
2. Trustworthiness					
Can be trusted/Cannot be trusted	4.31	1.448	.310	.743	.078
Accurate/Inaccurate	4.37	1.278	.149	.714	.036
Factual/Opinionated	4.25	1.511	.225	.700	.008
Concerned (not concerned) about the community's well-being	4.75	1.506	.150	.624	.067
Does (not) watch after reader's interests	4.35	1.416	.082	.609	.112
3. Dynamism					
Active/Passive	4.43	1.245	.061	.011	.727
Energetic/Tired	4.27	1.175	.169	.156	.718
Verbal/Quiet	4.20	1.194	.099	.167	.656
Bold/Timid	4.29	1.069	.028	.065	.634
Aggressive/Meek	3.86	1.277	.266	.088	.608

^*^ Based on 1 to 7 scales.

### Hypothesis Tests

Several preliminary tests were conducted before the hypotheses were tested. Omnibus analysis of variance (ANOVA) tests were run in order to detect unanticipated interaction effects between these hypothesized factors (domain and advertising) and the arthritis/depression topics. No three-way interactions emerged on any dependent variables, nor were there any two-way interactions involving the discussion topics. In order to address concerns about potential differences in scores due to the two data collection methods (paper-based vs Web-based), an additional ANOVA was conducted involving method, domains, and advertising. No significant three-way interactions or two-way interactions involving the hypothetical factors of interest obtained. The scores from paper-based version (*M* = 30.96, *SD* = 6.2) were somewhat higher on the *safety* dimension of credibility only than were scores obtained from the Web version of the same stimuli (*M* = 27.31, *SD* = 6.15), *t* (151) = 3.33, P = .001, but this main effect occurred across the board, and thus the findings reported below are not affected by data gathering method. The final analysis is based on the combined samples.

We predicted that different domains influence Web-site credibility (H1), and that both the *.com* domain and the presence of advertising reduce Web-site credibility (H2 and H3, respectively). Reduced, two-factor analyses were conducted involving domains and advertisements on the three dimensions of credibility.

On the *safety* dimension, ANOVA yielded a two-way interaction between domains and advertising, *F* (3, 145) = 2.73, P = .046, η^2^ = .05. The descriptive statistics for each cell are reported in Table 2. Inspection of the means indicated a disordinal interaction effect. Thus, no further main effects analyses were appropriate. The interaction indicates that for different domains, there were differences in perceived safety depending on the presence or absence of advertising.

**Table 2:** Impact of advertisement and domain on safety dimension

Ads Presence	Domain	*M*	*SD*	*n*
No ads	.org	32.60	5.66	20
	.com	28.70	6.29	20
	.edu	28.63	5.34	22
	.gov	31.32	7.05	19
With ads	.org	27.88	6.75	16
	.com	32.31	6.62	16
	.edu	28.58	5.48	21
	.gov	30.39	7.31	19

Most dramatically, the *.org* page received the highest mean when no advertising appeared, but when advertising was present, *.org* had the lowest mean, and the two versions were significantly different, *t* (34) = 2.29, P = .03. The *.com* site without advertising was among the lowest in safety, but just as low as both versions of the *.edu* site, which was low whether there was advertising or not. Interestingly, and contrary to hypotheses, the *.com* site with advertising on it was not the lowest rated among the versions that had advertising on them, although they were not significantly different using post hoc Newman Kuels tests. Both the *.gov* sites were both moderately high in safety. It is unclear which domain was seen as connoting the most safety; the *F* test seemed to obtain because of the difference between *.org* sites due to advertising.

On the *trustworthiness* dimension, the omnibus ANOVA revealed a two-way interaction effect also, *F* (3, 145) = 2.81, P = .041, η^2^ = .06. The pattern of the means was similar to that of the safety dimension. The *.org* page with advertising was the lowest scoring domain, but the same *.org* domain without advertising was the highest, *t* (34) = 2.80, P = .008. The scores for the .*edu* pages approached the scores of the *.com* with no advertising page; both were relatively low, whereas the *.gov* pages were relatively high on trustworthiness. The *.com* with advertising was not lower than *.com* without advertising; the difference between these two versions of *.com* was not statistically significant. However, among the pages that showed advertisements, the *.org* page was significantly lower in trustworthiness than the *.com* page as shown using Newman Kuels tests. Means and standard deviations for the two-way interaction are reported in Table 3.

**Table 3:** Impact of advertisement and domain on trustworthiness dimension

Ads Presence	Domain	*M*	*SD*	*n*
No ads	.org	23.50	4.30	20
	.com	22.45	5.35	20
	.edu	20.59	5.67	22
	.gov	23.11	5.88	19
With ads	.org	18.94	5.48	16
	.com	24.16	6.58	16
	.edu	22.10	4.86	21
	.gov	21.42	5.01	19

The *dynamism* dimension was not affected by any main or interaction effects across the board. However, between just the two *.edu* sites, the one without advertising (*M*= 19.59, *SD* = 3.96, *n* = 22) was significantly lower on dynamism than the *.edu* site with advertising, (*M* = 22.48, *SD* = 3.89, *n* = 21), *t* (41) = -2.41, P = .021, contradicting H3.

## Discussion

The present study sought to identify a parsimonious and appropriate set of measures to assess the way Internet users determine the credibility of health information online, and to examine some superficial yet common Web components that may affect credibility. While a plethora of measures and competing dimensions exist, past research has shown, and the present results reaffirm, that the measurement of credibility may shift because of the nature of the topic and other characteristics. In this research, scales drawn from a variety of potentially relevant sources and administered to adult samples resulted in a set of three dimensions, with similarity to dimensions found in previous research but unique with respect to the precise combination of scales.

Previous research conducted in non-health contexts has implicated domains and advertising as credibility variables. We hypothesized differences due to the domain of the site (H1), and this hypothesis was partially supported on two dimensions of credibility: *safety* and *dynamism*, but the effects were not straightforward. Specifically, for the *safety* dimension, the *domains* effect interacted with the presence or absence of advertising. Likewise, for *trustworthiness,* an interaction overrode main effects. Only on the *dynamism* dimension did domain and advertising not interact. However, there were no main effects on *trustworthiness* either.

There is also inconsistent support for our hypothesis that advertising has deleterious credibility effects (H3). Only in the cases of *.org* did trends go in this direction on two of the three dimensions of credibility: The opposite trends emerged, marginally for *.com*, and significantly (on *dynamism*) for *.edu*.

Findings differ from research on Web credibility in other domains, reaffirming the need to re-examine measurement in this context. In one sense, it confirms the criticism of the factor approach to source credibility that credibility perceptions may not be invariant or stable, but rather are sensitive to topic and context. It was not expected that the two predicted effects would interact, but the results indicate this is the case. This suggests that findings from research on one kind of Web site-non-health-related-may not generalize to other information contexts.

The *.com* domain was originally posited to elicit low credibility assessments because of the implied commercial self-interest of the site's sponsors. The .*com* domain elicited inconsistent responses, however. It may be that a commercial *.com* site without advertising may not appear as legitimate, ie, as deserving of additional commercial investment by means of advertising, as one in which advertisers have invested. Based on all the above findings, we notice that the effects of domains and advertising on Web-site credibility are not simple and straightforward. The domain and the presence of advertising are important factors in predicting the credibility of health Web sites, although they are important through their mutual interplay rather than individually.

These findings in particular sharply contrast with previous work on perceptions ofWeb-site credibility due to domain and advertising [30]. It is apparent from these results that the credibility of health-information presentations online is evaluated differently than previous findings on non-health Web sites.

It is also possible that the top-level domain of a site alone is not as important as it once was. If users find sites via search engines, the quality of the "hit" or search accuracy may be more important than the site's actual sponsor in most cases [31]. Users will probably be more likely to visit sites recommended by peers, and are more likely to find them credible, than sites they might find by other means. This is known to be the case with educationally useful site referrals among student peers [37].

The practical applications of this research are straightforward. When it is possible to choose the top-level domain for a health-information site, investing in an *.org* domain name appears to be worthwhile. Those affiliated with educational institutions, for whom an *.edu* Web site may be simple to create, are advised to establish an alternative. However, for those who must offset the costs of their efforts through online advertising, *.org* should be avoided. The credibility of other domains is not as strongly affected by the advertising decision.

Research applications may also be discerned from this investigation. Studies intended to test alternative forms of health information against Web-borne advice should include Web sites deliberately chosen on the basis of credibility, so that deficits in the persuasive aspects of alternative stimuli are not confused with the persuasive potential of the Web overall. Future research may broaden the question of how people are influenced by online health information, and compare the influence of source credibility to source homophily (ie, perceived similarity between source and user). Such comparisons hold promise for distinguishing the influence mechanisms that may differ between Web-site information and information exchanged through peer-to-peer support groups, harkening back to the distinction Hovland et al [10] made between expertise and trustworthiness as alternative and orthogonal sources of influence.

Finally, since credibility is not and end-goal in and of itself, but a facilitator of persuasion, attitude, and behavior, additional measures should be investigated that assess the likely adoption of information as a result of the two kinds of online presentations. In this regard, recent work by Dutta-Bergman [33] offers useful and validated scales for the evaluation and persuasiveness of online health information and its effect on attitude about, and intention toward, health-related behavior. The inclusion of such research methods will offer a more comprehensive and meaningful approach to a growing understanding of the impact of online health information in its various forms.

## Abbreviations

ANOVA: Analysis of Variance
